# Effects of Cardiac Sympathetic Neurodegeneration and PPAR*γ* Activation on Rhesus Macaque Whole Blood miRNA and mRNA Expression Profiles

**DOI:** 10.1155/2020/9426204

**Published:** 2020-05-02

**Authors:** Jeanette M. Metzger, Mary S. Lopez, Jenna K. Schmidt, Megan E. Murphy, Raghu Vemuganti, Marina E. Emborg

**Affiliations:** ^1^Preclinical Parkinson's Research Program, Wisconsin National Primate Research Center, University of Wisconsin–Madison, Madison, 53706 WI, USA; ^2^Cellular and Molecular Pathology Graduate Program, University of Wisconsin–Madison, Madison, 53706 WI, USA; ^3^Department of Neurosurgery, University of Wisconsin–Madison, 53706 Madison, WI, USA; ^4^Department of Medical Physics, University of Wisconsin–Madison, 53706 Madison, WI, USA

## Abstract

Degeneration of sympathetic innervation of the heart occurs in numerous diseases, including diabetes, idiopathic REM sleep disorder, and Parkinson's disease (PD). In PD, cardiac sympathetic denervation occurs in 80-90% of patients and can begin before the onset of motor symptoms. Today, there are no disease-modifying therapies for cardiac sympathetic neurodegeneration, and biomarkers are limited to radioimaging techniques. Analysis of expression levels of coding mRNA and noncoding RNAs, such as microRNAs (miRNAs), can uncover pathways involved in disease, leading to the discovery of biomarkers, pathological mechanisms, and potential drug targets. Whole blood in particular is a clinically relevant source of biomarkers, as blood sampling is inexpensive and simple to perform. Our research group has previously developed a nonhuman primate model of cardiac sympathetic denervation by intravenous administration of the catecholaminergic neurotoxin 6-hydroxydopamine (6-OHDA). In this rhesus macaque (*Macaca mulatta*) model, imaging with positron emission tomography showed that oral administration of the peroxisome proliferator-activated receptor gamma (PPAR*γ*) agonist pioglitazone (*n* = 5; 5 mg/kg daily) significantly decreased cardiac inflammation and oxidative stress compared to placebo (*n* = 5). Here, we report our analysis of miRNA and mRNA expression levels over time in the whole blood of these monkeys. Differential expression of three miRNAs was induced by 6-OHDA (mml-miR-16-2-3p, mml-miR-133d-3p, and mml-miR-1262-5p) and two miRNAs by pioglitazone (mml-miR-204-5p and mml-miR-146b-5p) at 12 weeks posttoxin, while expression of mRNAs involved in inflammatory cytokines and receptors was not significantly affected. Overall, this study contributes to the characterization of rhesus coding and noncoding RNA profiles in normal and disease-like conditions, which may facilitate the identification and clinical translation of biomarkers of cardiac neurodegeneration and neuroprotection.

## 1. Introduction

Cardiac function is modulated by the sympathetic and parasympathetic input of the autonomic nervous system. Sympathetic innervation to the heart is critical to regulate cardiac activity during exercise, changes in posture, and other nonpathological activities that alter blood pressure or heart rate [1–3]. Patients with diabetes [4, 5], REM sleep disorder [6], and Parkinson's disease (PD) frequently show loss of cardiac sympathetic innervation [7]. Sympathetic cardiac neurodegeneration occurs in 80-90% of PD patients, such that clinical guidelines now include *in vivo* imaging evidence of cardiac sympathetic denervation as a supportive diagnostic criterion [8–11].

Nonhuman primates are critical for modeling and studying diseases, due to their genetic and physiological similarity to humans [12]. Our research group has developed a rhesus macaque (*Macaca mulatta*) model of cardiac sympathetic denervation using intravenous administration of the neurotoxin 6-hydroxydopamine (6-OHDA) [13]. After entering neurons via catecholamine transporters, 6-OHDA autoxidizes and interferes with mitochondrial complex I, leading to increased oxidative stress and inflammation [14], which are neurodegenerative mechanisms shared with PD [15, 16]. We have shown that 6-OHDA-induced cardiac sympathetic loss, reactive oxygen species accumulation, and inflammatory cell recruitment can be detected by *in vivo* positron emission tomography (PET) [17]. Furthermore, administration of pioglitazone, a peroxisome proliferator-activated receptor gamma (PPAR*γ*) agonist, significantly reduced cardiac oxidative stress and inflammation [17]. PPAR*γ* is a type II nuclear receptor that classically works as a transcription factor by binding to PPAR*γ* response elements (PPREs) in the promoters of target genes [18], including coding and noncoding RNAs [19, 20]. PPAR*γ* activation inhibits the actions of nuclear factor kappa-light-chain-enhancer of activated B cells (NF-*κ*B), a transcription factor which increases immune cell expression of proinflammatory gene products including IL1*β*, TNF*α*, IL8, and IL6 [21, 22]. The cardiac PET findings and the transcriptional regulatory activity of PPAR*γ* suggest that the effects of systemic 6-OHDA and pioglitazone on the heart may be, at least in part, due to alterations of the transcriptome of inflammatory cells.

Transcriptome analysis has evolved from focusing on protein coding mRNA to include noncoding RNAs, as the latter are recognized as key regulators of protein expression and cell function. Noncoding RNAs include microRNAs (miRNAs), which are short, approximately 22-nucleotide RNAs. By binding to the 3′ untranslated region of target mRNAs, miRNAs inhibit translation of mRNAs into proteins. Each miRNA can target multiple mRNA sequences, affecting several functional pathways simultaneously [23]. miRNAs are key regulators of cellular health and disease progression [24] and can provide insight into disease mechanisms and serve as biomarkers. For example, analysis of whole blood samples has shown miRNA expression changes associated with renal sympathetic denervation to treat arterial hypertension [25], and circulating RNAs are an area of active research as potential biomarkers for PD [26–29]. However, alterations in mRNA and miRNA levels have not been studied in the context of cardiac sympathetic nerve loss in PD or animal models of PD. Additionally, our group is the first to evaluate miRNA expression in rhesus whole blood [30]. Here, we report our analysis of miRNA and mRNA expression levels over time in the whole blood of rhesus macaques with ongoing cardiac sympathetic neurodegeneration and PPAR*γ* activation.

## 2. Materials and Methods

### 2.1. Ethics Statement

The present study was performed in strict accordance with the recommendations in the National Research Council Guide for the Care and Use of Laboratory Animals (2011) in an AAALAC-accredited facility (Wisconsin National Primate Research Center, University of Wisconsin-Madison). Experimental procedures were approved by the Institutional Animal Care and Use Committee (IACUC) of the University of Wisconsin-Madison (permit number: G00705). All efforts were made to minimize the number of animals used and to ameliorate any distress.

### 2.2. Subjects and Whole Blood Collection

Blood samples for this project were obtained from ten adult, male rhesus macaques treated with systemic 6-OHDA (50 mg/kg IV, [13]) that were part of a previously published study [17]. Twenty-four hours post-6-OHDA, animals were randomly assigned to receive daily oral dosing of placebo (*n* = 5; 6.2–13.0 years old; 9.8–12.3 kg) or PPAR*γ* agonist pioglitazone (5 mg/kg; *n* = 5; 5.6–11.4 years old; 9.4–10.6 kg). Blood samples were collected in PAXgene blood RNA tubes (Qiagen, Venlo, Netherlands) under ketamine anesthesia (15 mg/kg IM) before, 1 week after, and 12 weeks after 6-OHDA and stored at -80°C until RNA extraction. All 30 samples were randomized and arbitrarily numbered to achieve blinding.

### 2.3. RNA Isolation

Total RNA was extracted following 24 hr incubation at room temperature. Immediately after isolation, RNA quantity and quality were assessed using NanoDrop 2000 (Thermo Fisher Scientific, Halethorpe, MD, USA) and Bioanalyzer 2100 (Agilent, Santa Clara, CA, USA) (Supplementary Table 1). Aliquots of RNA were stored at -80°C.

### 2.4. miRNA Library Preparation and Next-Generation Sequencing (NGS)

Library preparation, NGS, and data analysis were performed by Qiagen Genomic Services (Frederick, Maryland). Additionally, Qiagen Genomic Services assessed nucleic acid quality before and after library preparation using Bioanalyzer 2100 and TapeStation 4200 (Agilent, Santa Clara, CA, USA). The miRNA sequencing libraries were prepared with the QIAseq miRNA Library Kit (Qiagen, Venlo, Netherlands) using 100 ng total RNA. Adapters containing unique molecular indices (UMIs) were ligated to the RNA. miRNAs and small RNAs (e.g., small nucleolar RNAs (snoRNAs) and small nuclear RNAs (snRNAs)) were converted to cDNA amplified using PCR (16 cycles). Amplified RNAs were sequenced using the NextSeq 500 (Illumina, San Diego, CA, USA) generating single-end reads with a sequencing read length of 76 nucleotides. Raw data was converted to FASTQ files using the bcl2fastq software and checked using the FastQC tool. Reads with identical UMIs were collapsed into a single read and aligned to miRBase using Bowtie2 to identify known miRNAs in rhesus macaques to generate miRNA counts. The reference genome used was Mmul_1 and annotation reference with miRBase_20. Small RNAs are reads that align to the Qiagen small RNA database. Unmapped reads that did not align with rhesus or other species in miRBase were analyzed by the miRPara tool to identify putative miRNAs [31]. RNA read counts were normalized for sample sequencing depth by calculating tags per million (TPM), the number of reads for a particular miRNA in an individual subject divided by the total number of mapped reads in that subject and multiplied by 1 million.

### 2.5. mRNA RT^2^ Profiler PCR Array and Additional Gene Reverse Transcription-Quantitative PCR (RT-qPCR)

RT-qPCR was used to assess genes included on Inflammatory Cytokines and Receptors RT^2^ Profiler PCR Array (Qiagen, Venlo, Netherlands) (Supplementary Table 2) and additional genes of interest (*NFKBIA*, *CD36*, *STAT1*, and *MAFB*) (Supplementary Table 3). 5 *μ*g of total RNA was synthesized into cDNA using the RT^2^ First Strand Kit (Qiagen, Venlo, Netherlands) according to the manufacturer's instructions and then either used immediately or stored at -80°C. All qPCR was carried out on the Applied Biosystems QuantStudio 3 (Thermo Fisher, Waltham, MA, USA) using RT^2^ SYBR Green ROX qPCR Mastermix (Qiagen, Venlo, Netherlands). All control wells in the PCR arrays were within expected cycles to threshold (Cts). Additional gene qPCR was performed on 96-well plates with each gene of interest on the same plate as the reference gene, each sample in duplicate, and the inclusion of no template control wells for each gene and the reference gene. Duplicate wells for each sample were averaged for data analysis. The Ct of all no template control wells was >35. *NFKBIA*, *CD36*, *STAT1*, and *MAFB* primers were designed using the PrimerQuest online tool (Integrated DNA Technologies); qPCR products were loaded on 2% agarose gels, and amplification of a single band of the predicted size was verified for each primer pair (Supplementary Figure 1). Fold differences in gene expression were calculated using the 2^−*ΔΔ*Ct^ fold change method and presented as fold change [32]. Cts were normalized to the reference gene *RPL13A*; primers for *RPL13A* were included on the PCR arrays, and the same primers were purchased for additional gene RT-qPCR (Product no. 33001; Cat. no. PPQ00210B-200). This gene was selected based on having the most stable expression across all samples and previous results illustrating *RPL13A* as a suitable reference gene specifically in rhesus macaques [33].

### 2.6. Data Analysis

miRNA or other small RNA differential expression analysis was carried out using edgeR (Bioconductor). For normalization, the trimmed mean of *M* value method based on log-fold and absolute gene-wise changes in expression levels between samples (TMM normalization) was used. All *p* values were corrected for multiple corrections using the false discovery rate (FDR) correction method of Benjamini and Hochberg. RNAs were considered significantly differentially expressed when the FDR-corrected *p* value was <0.05 and fold change was >|2|. Principal components analysis (PCA) was performed with R using TMM-normalized quantifications. PCA plots identified one placebo group animal (Placebo 3) as a possible outlier (Supplementary Figure 2). Differential expression analysis was performed with and without the outlier (Supplementary Table 4; Supplementary Table 5; Supplementary Table 6); results from the analysis without the outlier are reported and discussed. miRBase version 22.1 was used for comparison between the rhesus macaque (mml-), human (hsa-), mouse (mmu-), and rat (rno-) mature miRNA (miR) and miRNA precursor (mir) sequences.

mRNA gene targets of miRNAs were identified using TargetScan (v7.2) with species selected as “Rhesus” (http://www.targetscan.org). Enrichr (http://amp.pharm.mssm.edu/Enrichr/) [34, 35] was used to perform Gene Ontology (GO) function annotation and Kyoto Encyclopedia of Genes and Genomes (KEGG) biological pathway analyses. The Enrichr ontology functions analyzed were GO Biological Process 2018, GO Molecular Function 2018, and GO Cellular Component 2018. The Enrichr pathway function analyzed was KEGG 2016. GO and KEGG term *p* values were calculated using the Fisher exact and corrected for multiple comparisons using FDR. Functional enrichment analysis was limited to mml-miR-16-2-3p as it was the most abundant differentially expressed miRNA (showed high expression levels (>10 TMM) in all groups) and exhibited the highest fold change between groups.

mRNA data was analyzed for differential expression using two-way repeated-measures analysis of variance (ANOVA) in SPSS (version 24). Ct values > 35 were replaced with 35 prior to calculating *Δ*Ct for data analysis. All *p* values were corrected for multiple comparisons using FDR (http://www.sdmproject.com/utilities/?show=FDR); uncorrected and FDR-corrected *p* values are reported. Volcano plots were constructed in R (version 3.5.1).

## 3. Results and Discussion

### 3.1. NGS Overview

NGS detected an average of 10.3 million UMI-corrected reads in each library (Supplementary Table 7) at a high base quality (Supplementary Figure 3). Averaging the data from all 30 samples, 85.86% of UMI-corrected reads were mapped to known rhesus macaque miRNAs, 0.29% were mapped to small RNAs other than miRNAs, and 10.04% were aligned to the rhesus reference genome, but not to miRNA or small RNAs (Supplementary Table 7). Additionally, 0.14% of the reads represented miRNAs not before documented in rhesus macaques or putative miRNAs that did not map to any species in miRBase; a description of these novel miRNA findings has been recently published by our group [30].

### 3.2. miRNA

#### 3.2.1. miRNA Expression in Rhesus Macaque Whole Blood

**Figure 1:**
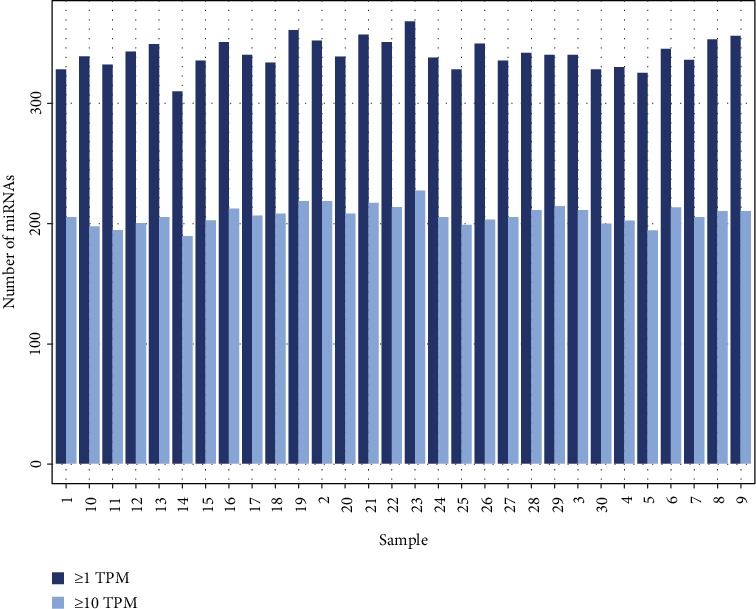
Bar graph illustrating the number miRNAs observed at ≥1 or 10 TPM (Tags Per Million) in each sample. TPM is equal to the number of reads for a particular miRNA in an individual subject divided by the total number of mapped reads in that subject, multiplied by 1 million. 266 miRNAs were identified in all samples at ≥1 TPM; 158 miRNAs were identified in all samples at ≥10 TPM.

Of the 912 known rhesus mature miRNAs, 419 were observed in the whole blood in at least one sample of this study above a threshold of 1 TPM (Supplementary Table 8). A total of 266 and 158 miRNAs were detected at 1 TPM or 10 TPM, respectively, in all 30 of the samples (Figure 1). miRNA expression is known to show large variability between tissues [36, 37], likely explaining why this study detected approximately 50% of the known rhesus miRNome.

**Figure 2:**
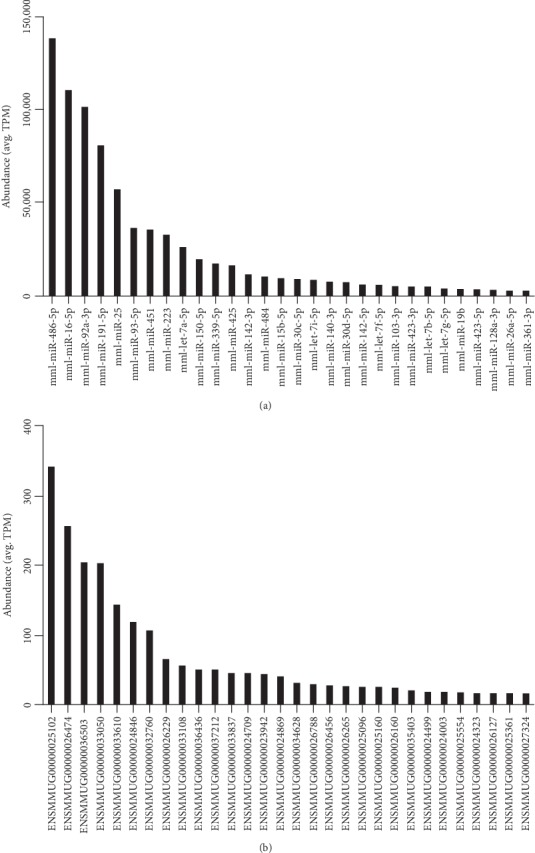
The top 30 most abundant (a) mature miRNAs and (b) small RNAs in the whole blood of normal rhesus macaques. Abundance is given as the average (mean) tag per million (TPM) for each RNA in 10 rhesus at baseline.

In naïve, baseline samples representative of the whole blood miRNA profile in normal rhesus, mml-miR-486-5p, mml-miR-16-5p, mml-miR-92a-3p, mml-miR-191-5p, and mml-miR-25 were the top 5 most abundant mature miRNAs and represented over 50% of total miRNA reads (Figure 2(a)). These findings are similar to other species, including humans [38] and rats [39], and support the concept that these miRNAs are conserved between species (Table 1). In humans, hsa-miR-16-5p, hsa-miR-486-5p, and hsa-miR-92a constitute 20.6%, 12.6%, and 12.6% of the mature miRNAs in red blood cells, respectively [40]. Our results in rhesus suggest that, like humans [41, 42], the whole blood miRNA expression pattern reflects red blood cells' transcriptome more than leukocytes.

#### 3.2.2. miRNA Differential Expression

**Figure 3:**
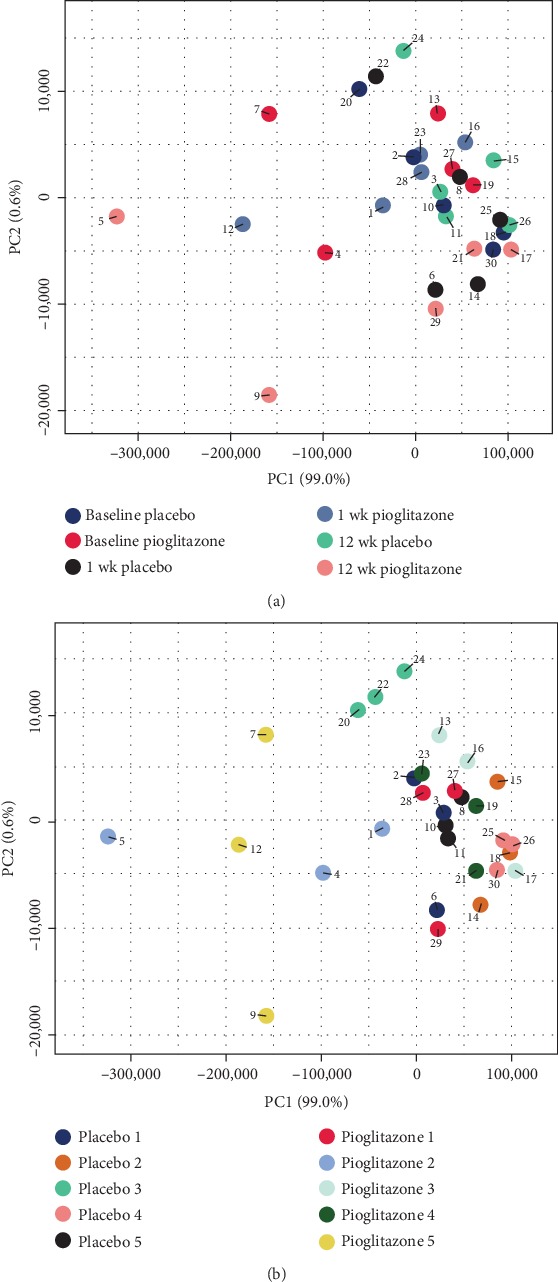
Principal components analysis (PCA) plots showing unsupervised assessment of all 30 samples. Data points are colored by (a) experimental group or (b) individual subject. Note that samples cluster based on subject (b) but are spread out in treatment groups (a). The PCA was performed on all samples using the 50 miRNAs with the largest coefficient of variation based on TMM-normalized counts. Each circle represents one sample. 1wk, 1 week post-6-OHDA; 12wk, 12 weeks post-6-OHDA.

Unsupervised PCA analysis indicated that each individual animal exhibited a unique miRNA profile that was stable over time, as demonstrated by samples clustering closely based on the individual (Figure 3(b)) but not treatment (Figure 3(a)). Relative to inbred rodent strains, outbred research species such as rhesus macaques exhibit high individual genetic variability [43]. This genetic variability produces individual differences in RNA expression levels [44], likely contributing to the sample clustering pattern observed in PCA (Figure 3). Despite this, five miRNAs were detected which had statistically significant changes in expression levels following systemic 6-OHDA or PPAR*γ* activation (Table 2), including mml-miR-16-2-3p, mml-miR-204-5p, mml-miR-133d-3p, mml-miR-1262-5p, and mml-miR-146b-5p.

Systemic 6-OHDA increased the expression of mml-miR-16-2-3p and decreased mml-miR-133d-3p and mml-miR-1262-5p 12 weeks posttoxin, identifying these miRNAs as potential biomarkers of the long term (12 week) effects of sympathetic denervation. No miRNAs were found to be differentially expressed at the 1 week timepoint. The impact of increased mml-miR-16-2-3p levels 12 weeks posttoxin is unclear. Although mml-miR-16-2-3p was predicted to have over 3000 mRNA targets (Supplementary Table 9), this list of targets was not significantly enriched for mRNAs involved in any specific biological pathway defined in the KEGG database (Supplementary Table 10). The 16 GO terms significantly enriched for miR-16-2-3p gene targets were categorized into 12 biological processes and 4 molecular functions, including ubiquitin ligase activity and regulation gene transcription and expression (Table 3; Supplementary Table 11). These functions suggest that mml-miR-16-2-3p affects a broad range of cellular pathways and could have wide-reaching effects on multiple cell types. The few studies available on miR-16-2-3p have linked it to cancers [45, 46], craniofacial malformations [47, 48], treatment of PD with Levodopa/Carbidopa [49], and multiple sclerosis [50], supporting diverse roles for this miRNA. Future studies investigating the blood cell type(s), or noncellular sources such as exosomes [51, 52], in which mml-miR-16-2-3p expression is altered by 6-OHDA will give insight into the relationship between sympathetic denervation and mml-miR-16-2-3p.

Information about mml-miR-133d-3p and mml-miR-1262-5p is also limited. Mml-miR-133d-3p is now recognized to be mml-miR-133a in the current miRBase version 22.1 following the release of the rhesus macaque Mmul_8.0.1 genome assembly. Mml-miR-133a contains a different mRNA binding seed sequence, the nucleotides in the 2-7 positions at the 5′ end of the miRNA, than the most similar human sequence, hsa-miR-133a-3p (Table 4). The rhesus mml-miR-1262-5p sequence is dissimilar from the human hsa-miR-1262, and a search using miRBase does not indicate an equivalent miRNA sequence in humans. Publications are not currently available on the function of mml-miR-133d-3p and mml-miR-1262-5p in rhesus, and the dissimilarity with human miRNA sequences cautions against comparison with reports in humans. The function of these RNAs will hopefully be elucidated as new studies on miRNAs in rhesus macaques emerge.

PPAR*γ* activation by pioglitazone led to increased expression of mml-miR-146b-5p from baseline to 12 weeks post-6-OHDA, in addition to decreased levels of mml-miR-16-2-3p and mml-miR-204-5p in pioglitazone-treated animals relative to placebo-treated at the twelve-week time point. In this context, these miRNAs emerge as potential biomarkers of the long term (12 week) effects of PPAR*γ* activation. PPAR*γ* activation in immune cells is well documented to downregulate activity of the transcription factor NF-*κ*B and downstream pro-inflammatory cytokines and chemokines [21, 22]. Fitting this function, miR-204-5p and miR-146b-5p are involved in inflammation. In mice, mmu-miR-204-5p is upregulated in pro-inflammatory type macrophages relative to anti-inflammatory macrophages [53]. Downregulation of mml-miR-204-5p in the current study may reflect a PPAR*γ*-associated skewing of circulating monocytes to an anti-inflammatory phenotype. Interestingly, the opposite effect was found in human pulmonary arterial smooth muscle cells, in which hsa-miR-204-5p expression decreased following repression of PPAR*γ* [54], suggesting a differential effect of PPAR*γ* by cell type. Future work investigating the function of miR-204-5p in blood cells and pulmonary arterial smooth muscle with be important to understanding the impact of altered miR-204-5p expression in these cell types.

In humans, hsa-miR-146-5p has been identified as a critical regulator of the immune response, as well as in the pathophysiology of several cancers, renal, and cardiac diseases [55, 56]. Hsa-miR-146-5p exerts an anti-inflammatory effect by decreasing expression of proteins such as TNF receptor-associated factor 6 (TRAF6), a signal transducer upstream of NF-*κ*B activation [57–59]. Our findings of differential expression of mml-miR-146b-5p and mml-miR-204-5p suggest a relationship between these miRNAs and the anti-inflammatory actions of pioglitazone observed in the heart of these animals [17]. In contrast to our findings, pioglitazone administration led to decreased levels of rno-miR-146b-5p in the rat heart in a model of heart failure; this result followed an increase rno-miR-146b-5p associated with the induction of heart failure in the model [60]. Additional research will be needed to shed light on whether these differential effects of pioglitazone on miR-146-5p expression levels are due to differences between species, tissues examined (cardiac vs. whole blood), or another factor. To the authors' knowledge, the present study is the first to report alterations of miRNAs in whole blood following pioglitazone administration. One publication describing miRNA alterations in serum found that hsa-miR-24 levels were increased in diabetic patients following 9 months of pioglitazone [61]; miR-24 expression was not impacted in the present study. Additional work is needed to assess replicability of differentially expressed miRNAs across studies in order to identify them as reliable biomarkers.

Although in this study 6-OHDA was used to model cardiac sympathetic neurodegeneration, it is important to recognize that the neurotoxin produces sympathetic denervation outside of the heart, including spleen and bone marrow. Peripheral sympathectomy with systemic 6-OHDA is reported to alter immune cell development and phenotype, leading to complex effects on the immune response [62–65]. Whole blood miRNA changes in our monkeys may be related to the direct effects of sympathetic denervation of lymphoid organs, rather than inflammation induced by cardiac neurodegeneration. As qPCR validation of differentially expressed miRNAs was not performed in this study due to limited sample availability, candidate biomarkers should be further validated in future studies.

### 3.3. Other Small RNAs

#### 3.3.1. Other Small RNA Expression in Rhesus Macaque Whole Blood

Small RNAs other than miRNAs identified by NGS included snoRNAs and snRNAs (Supplementary Table 5; Supplementary Table 6; Supplementary Table 8). A total of 85 small RNAs were present in all 30 samples at at least 1 TPM and 16 were present in all samples at at least 10 TPM (Supplementary Table 8). Of the 251 small RNAs detected at at least 1 TPM in any sample, 203 were snoRNAs and 48 were snRNAs (Supplementary Table 8). The QIAseq miRNA library kit used for the NGS portion of this study was developed primarily for miRNAs [66], thus the results may not reflect the full profile of snoRNAs and snRNAs in the whole blood of rhesus macaques.

In naïve, baseline samples, the top 5 most abundant snoRNAs or snRNAs detected in this study based on average TPM were the snoRNAs SNORND104 (ENSMMUG00000025102), SNORD83 (ENSMMUG00000026474), SCARNA3 (ENSMMUG00000036503), SNORD69 (ENSMMUG00000033050), and SNORD100 (ENSMMUG00000033610), which accounted for 40% of reads (Figure 2(b); Supplementary Table 8). The cellular function of snoRNAs is an area of active research [67] with limited information currently available [68–70]. Canonically, snoRNAs contain sequences complementary to ribosomal RNAs (rRNAs), which guide ribonucleoproteins necessary for rRNA modification such as 2-O'-methylation and pseudouridylation [67, 71].

#### 3.3.2. Other Small RNA Differential Expression

At 12 weeks post-6-OHDA, the snoRNA SNORA46 was significantly downregulated in the 6-OHDA + Pioglitazone group relative to 6-OHDA + Placebo (Table 2). 6-OHDA + Pioglitazone animals exhibited a significant increase in expression of SNORD15 from baseline to 12 weeks posttoxin (Table 2). No snoRNAs or snRNAs were found to be differentially expressed 1 week post-6-OHDA. SNORA46 is predicted to guide the modification of uridine (U469) in ribosomal 18S rRNA [72]. SNORA15, also known as U15, is predicted to guide the modification of adenosine (A3764) in 28S rRNA [70]. Currently, only one report exists of SNORA46 or SNORD15 involvement in human disease, showing auto-antibodies to SNORD15 in patients with systemic sclerosis [73]. The functional impact of altered snoRNA expression following systemic 6-OHDA or PPAR*γ* activation is unclear due to limited research on the effect of the above rRNA modifications on cellular activity.

### 3.4. mRNA

**Figure 4:**
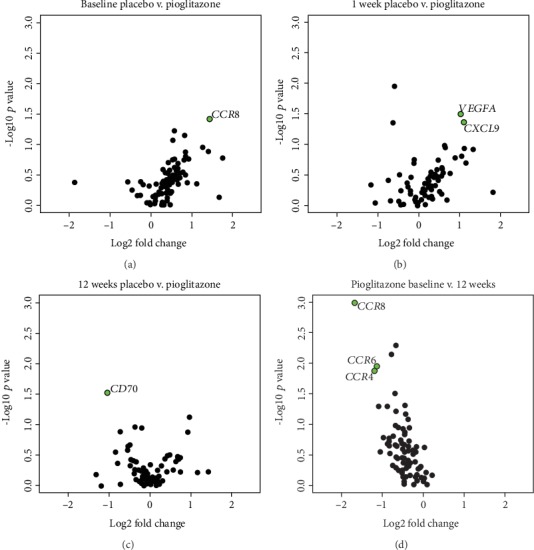
Volcano plots of differentially expressed mRNAs using uncorrected *p* values. Volcano plots illustrating differential expression between the 6-OHDA + Placebo and 6-OHDA + Pioglitazone groups at (a) baseline, (b) 1 week post-6-OHDA, (c) 12 weeks post-6-OHDA and (d) between the baseline and 12 weeks post-6-OHDA timepoints in the 6-OHDA + Pioglitazone group. Each circle represents one gene. Green filled circles represent genes for which the uncorrected *p* value is <0.05 and Log2 fold change is >1. Note that positive Log2 fold change indicates higher expression in the group to the right in the header (e.g., CCR8 showed higher expression in the 6-OHDA + Pioglitazone group at baseline) and negative Log2 fold change indicates higher expression in the group to the left. *CCR8*, C-C motif chemokine receptor 8; *VEGFA*, vascular endothelial growth factor A; *CXCL9*, C-X-C motif chemokine ligand 9; *CD70*, CD70 molecule; *CCR6*, C-C motif chemokine receptor 6; *CCR4*, C-C motif chemokine receptor 4.

Analysis of mRNA expression levels in whole blood suggested some differences between groups (Figure 4). However, correction of *p* values for multiple comparisons using the FDR correction revealed that no genes assessed in this study were significantly differentially expressed at the 1 week or 12 week time points (Supplementary Table 12; Supplementary Table 13). This lack of change in expression levels of inflammatory cytokine and receptor genes in whole blood is in contrast to the significant 6-OHDA-induced increase in cardiac inflammation and oxidative stress and the pioglitazone-associated amelioration of these neurodegenerative mechanisms observed 1 week post-6-OHDA by PET [17]. Pioglitazone also led to increased CD36 protein expression in the heart in these animals as detected by immunohistochemistry [74], but CD36 transcript levels in the blood were not found to be altered. These findings likely reflect differences in how the treatments affected cells in the heart compared to cells in the blood and also the unique transcriptional profiles of these tissues. Additionally, three differentially expressed miRNAs in this study, mml-miR-16-2-3p, mml-miR-133d-3p, and mml-miR-1262-5p, were predicted to target over 30 mRNAs assayed by PCR array and additional RT-qPCR (Supplementary Table 14), yet our results did not identify altered expression of these target mRNAs.

The lack of significant treatment effect on mRNA expression levels in our study may be related to the use of whole blood as the source of the RNA samples. Whole blood sampling was selected to maximize clinical relevancy. Whole blood samples are easy and inexpensive to collect, and the PAXgene tubes used in this study are FDA-cleared and CE-marked for in vitro diagnostic use. The use of whole blood RNA, however, can present technical challenges. Globin mRNA can represent 50-80% of mRNA transcripts within the total RNA extracted from whole blood, lowering sensitivity [75, 76]. Although methods are available to reduce globin mRNA content, these additional steps may increase sample variation, alter RNA profiles, and reduce RNA yield [77–79]. Previous reports have shown that pioglitazone significantly altered mRNA expression in human peripheral blood mononuclear cells (PBMCs) [80, 81]. Future work in this rhesus 6-OHDA model investigating mRNA changes in globin reduced whole blood RNA or in RNA extracted from PBMCs might have increased sensitivity to detect alterations in the inflammatory cytokine and receptor genes assessed in the present study.

## 4. Conclusions

At twelve weeks postneurotoxin, differential expression of three miRNAs was induced by 6-OHDA (mml-miR-16-2-3p, mml-miR-133d-3p, and mml-miR-1262-5p) and two miRNAs by pioglitazone (mml-miR-204-5p and mml-miR-146b-5p), providing initial evidence that these RNAs are circulating markers of the long term (12 week) effects of sympathetic denervation and PPAR*γ* activation, respectively. Expression of inflammatory cytokine and receptor mRNAs in rhesus macaque whole blood was unaffected by systemic 6-OHDA and PPAR*γ* activation.

Overall, this study contributes to the characterization of rhesus coding and noncoding RNA profiles in normal and disease-like conditions, which may facilitate the identification and clinical translation of biomarkers of cardiac neurodegeneration and neuroprotection.

## Figures and Tables

**Table 1:** Conservation between rhesus macaque, human, and rat miRNA sequences of miRNAs highly abundant in whole blood. The bold underscore in the rno-miR-92a-3p sequence indicates that the 3′ end of this sequence ends at ‘g' and does not include the ‘u' in the rhesus macaque and human sequences.

Highly abundant rhesus macaque whole blood miRNAs and most similar human and rat miRNAs	miRNA sequence (5′ to 3′)
mml-miR-486-5p	uccuguacugagcugccccgag
hsa-miR-486-5p	uccuguacugagcugccccgag
rno-miR-486-5p	uccuguacugagcugccccgag

mml-miR-16-5p	uagcagcacguaaauauuggcg
hsa-miR-16-5p	uagcagcacguaaauauuggcg
rno-miR-16-5p	uagcagcacguaaauauuggcg

mml-miR-92a-3p	uauugcacuugucccggccugu
hsa-miR-92a-3p	uauugcacuugucccggccugu
rno-miR-92a-3p	uauugcacuugucccggccug**_**

mml-miR-191-5p	caacggaaucccaaaagcagcug
hsa-miR-191-5p	caacggaaucccaaaagcagcug
rno-miR-191a-5p	caacggaaucccaaaagcagcug

mml-miR-25	cauugcacuugucucggucuga
hsa-miR-25-3p	cauugcacuugucucggucuga
rno-miR-25-3p	cauugcacuugucucggucuga

**Table 2:** Differentially expressed small RNAs. Results shown here represent differential expression analysis without the outlier animal (Placebo 3). TMM, trimmed mean of M values; snoRNA, small nucleolar RNA; miRNA, micro RNA; FDR, false discovery rate.

Groups compared (Group A vs. Group B)	Differentially expressed RNA	RNA type	Group A TMM	Group B TMM	Fold change	Uncorrected *p* value	FDR corrected *p* value
12 weeks Placebo vs. pioglitazone	mml-miR-16-2-3p	miRNA	371.46	69.25	-5.36	0.0001555	0.0374679
	mml-miR-204-5p	miRNA	35.53	9.53	-3.72	0.0003195	0.0384989
	*SNORA46* (ENSMMUG00000025257)	snoRNA	699.51	169.81	-3.81	0.0000211	0.0127114

Placebo Baseline vs. 12 weeks	mml-miR-16-2-3p	miRNA	114.74	326.76	2.31	0.0000004	0.0001542
	mml-miR-133d-3p	miRNA	40.33	8.28	-3.32	0.0000052	0.0009051
	mml-miR-1262-5p	miRNA	4.41	1.55	-2.58	0.0000467	0.0053706

Pioglitazone Baseline vs. 12 weeks	mml-miR-146b-5p	miRNA	4.54	9.70	2.20	0.0000418	0.0153731
	*SNORD15* (ENSMMUG00000026536)	snoRNA	1.70	5.80	2.42	0.0000004	0.0003415

TMM: trimmed mean of *M* values; snoRNA: small nucleolar RNA; miRNA: microRNA; FDR: false discovery rate.

**Table 3:** GO enrichment analysis of mml-miR-16-2-3p target genes. Note that no Cellular Component GO terms were statistically significant. GO, gene ontology.

	Significant GO enrichment category	Number of mml-miR-16-2-3p target genes in the enrichment category	FDR adjusted *p* value
Biological process	Regulation of transcription, DNA-templated (GO:0006355)	367	0.00000019832
	Regulation of gene expression (GO:0010468)	246	0.00001071521
	Regulation of cellular macromolecule biosynthetic process (GO:2000112)	162	0.00001511050
	Nervous system development (GO:0007399)	122	0.00006220983
	Regulation of nucleic acid-templated transcription (GO:1903506)	153	0.00008303183
	Regulation of transcription from RNA polymerase II promoter (GO:0006357)	324	0.00008778663
	Positive regulation of transcription, DNA-templated (GO:0045893)	246	0.00241166240
	Positive regulation of transcription from RNA polymerase II promoter (GO:0045944)	193	0.00249008012
	Transcription from RNA polymerase II promoter (GO:0006366)	120	0.00297232330
	Negative regulation of transcription, DNA-templated (GO:0045892)	185	0.00319111007
	Protein phosphorylation (GO:0006468)	112	0.02744139610
	Protein modification by small protein conjugation (GO:0032446)	97	0.03163906572

Molecular function	Ubiquitin-like protein ligase activity (GO:0061659)	55	0.01261271587
	Ubiquitin protein ligase activity (GO:0061630)	54	0.01261271587
	Ubiquitin-protein transferase activity (GO:0004842)	100	0.01261271587
	Integrin binding (GO:0005178)	31	0.03275663844

GO: gene ontology.

**Table 4:** Conservation between rhesus macaque and human miRNA sequences of differentially expressed miRNAs. Note that mml-miR-133d-3p is now recognized as mml-miR-133a. Italic and bold letters indicate nucleotides that differ between the rhesus and most similar human miRNA sequences. The bold underscore in the hsa-miR-133a-3p sequence indicates that the 3′ end of this sequence ends at ‘g' and does not include the ‘u' in the rhesus macaque sequence. n/a, not applicable.

Differentially expressed rhesus miRNAs and most similar human miRNAs	miRNA sequence (5′ to 3′)
mml-miR-16-2-3p	ccaauauuacugugcugcuuca
hsa-miR-16-2-3p	ccaauauuacugugcugcuu***u***a

mml-miR-204-5p	uucccuuugucauccuaugccu
hsa-miR-204-5p	uucccuuugucauccuaugccu

mml-miR-133d-3p	uugguccccuucaaccagcugu
hsa-miR-133a-3p	***u***uugguccccuucaaccagcug**_**

mml-miR-1262-5p	uucuauaaauucauccaucaca
n/a	n/a

mml-miR-146b-5p	ugagaacugaauuccauaggcu
hsa-miR-146b-5p	ugagaacugaauuccauaggcu***g***

## Data Availability

The data used to support the findings of this study are available in the supplementary materials or from the corresponding author upon reasonable request.

## Supplementary Materials

Supplementary 1 Supplementary Figure 1: gel electrophoresis of PCR products. Supplementary Figure 2: principal component analysis plots showing unsupervised assessment of placebo group samples. Supplementary Figure 3: average base quality of the UMI-corrected reads in each of the 30 RNA samples.

Supplementary 2 Supplementary Table 1: quality and concentration of all 30 extracted total RNA samples.

Supplementary 3 Supplementary Table 2: list of genes on the RT^2^ 475 rhesus inflammatory cytokine and receptor PCR array.

Supplementary 4 Supplementary Table 3: sequences of primers used for RT-qPCR.

Supplementary 5 Supplementary Table 4: differentially expressed small RNAs.

Supplementary 6 Supplementary Table 5: results of small RNA differential expression analysis between groups with all animals included (*n* = 10).

Supplementary 7 Supplementary Table 6: results of small RNA differential expression analysis between groups without the outlier animal (*n* = 9).

Supplementary 8 Supplementary Table 7: summary of sequencing reads and mapping for each sample.

Supplementary 9 Supplementary Table 8: abundance of miRNAs and other small RNAs.

Supplementary 10 Supplementary Table 9: mRNA targets of mml-miR-16-2-3p predicted by TargetScan.

Supplementary 11 Supplementary Table 10: results of Kyoto Encyclopedia of Genes and Genomes (KEGG) pathway analysis.

Supplementary 12 Supplementary Table 11: results of gene ontology (GO) enrichment analysis.

Supplementary 13 Supplementary Table 12: list of uncorrected *p* values, FDR-corrected *p* values, and fold change for all genes on the rhesus inflammatory cytokine and receptor RT^2^ profiler array.

Supplementary 14 Supplementary Table 13: list of uncorrected *p* values, FDR-corrected *p* values, and fold change for additional gene qPCR.

Supplementary 15 Supplementary Table 14: predicted mRNA targets of mml-miR-16-2-3p, mml-miR-204-5p, mml-miR-133d-3p, mml-miR-1262-5p, and mml-miR-146b-5p.
