# Supplementary material for: Effects of Cardiac Sympathetic Neurodegeneration and PPARγ Activation on Rhesus Macaque Whole Blood miRNA and mRNA Expression Profiles
Source: Biomed Res Int. 2020 May 2;2020:9426204. doi: 10.1155/2020/9426204 (PMC7212295; doi:10.1155/2020/9426204)
Supplement: Supplementary 1 — Supplementary Figure 1: gel electrophoresis of PCR products. Supplementary Figure 2: principal component analysis plots showing unsupervised assessment of placebo group samples. Supplementary Figure 3: average base quality of the UMI-corrected reads in each of the 30 RNA samples. [file 9426204.f1.docx]

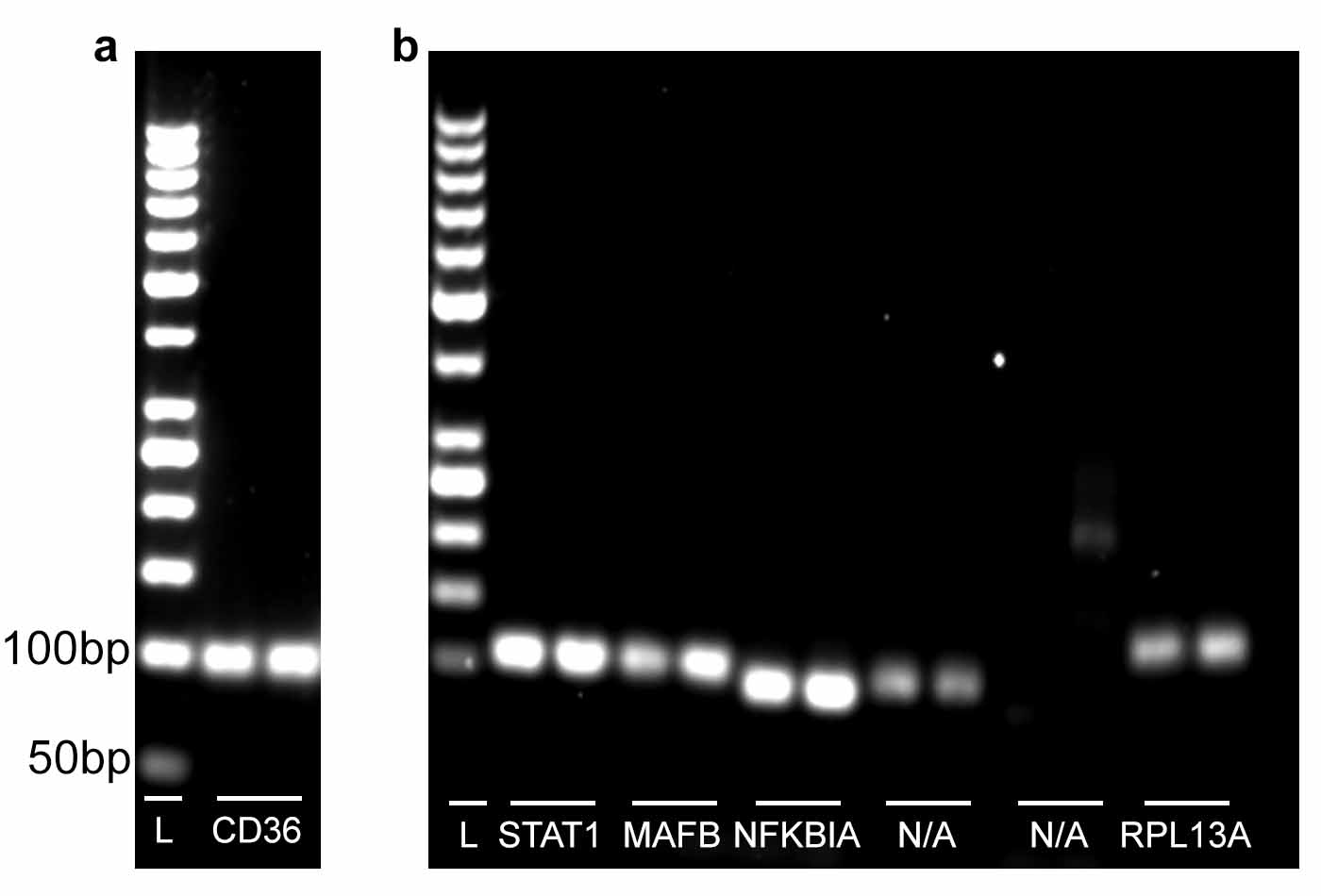


Supplementary Figure 1. Gel electrophoresis of PCR products illustrating a single band was produced for the primers used for additional gene RT-qPCR. Note that small differences in the appearance of the ladder bands between (a) and (b) are related to difference in gel composition (3% agarose in ‘a’ and 2% agarose in ‘b’) and smaller volume of ladder used in (b) relative to (a). Amplicon lengths: CD36, 97bp; STAT1, 101bp; MAFB, 102bp; NFKBIA, 91bp; RPL13A, 121bp. L, ladder; bp, base pairs; N/A, not applicable (primer set not used for this study).


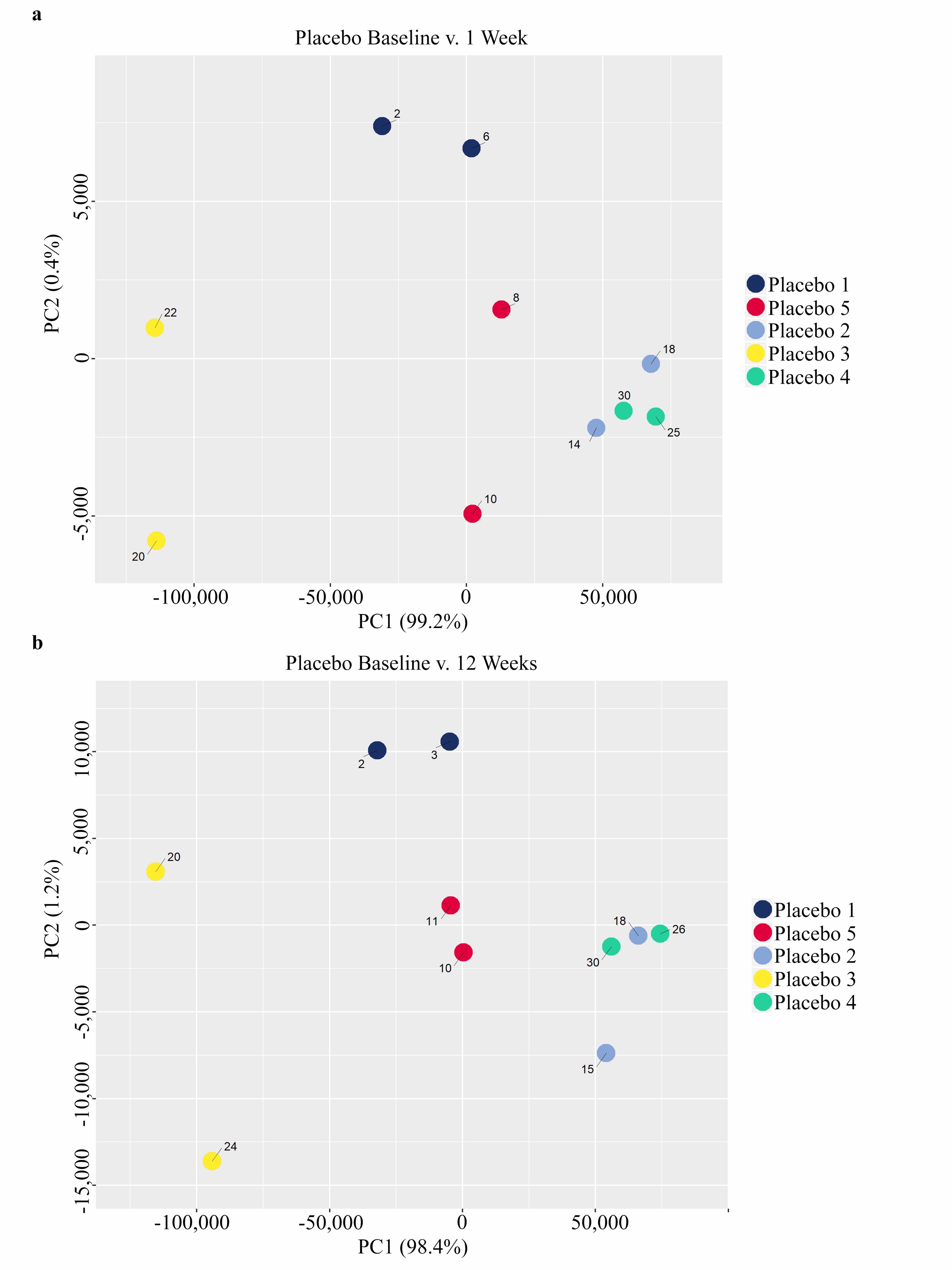


Supplementary Figure 2. Principal components analysis (PCA) plots showing unsupervised assessment of Placebo group samples. (a) Includes baseline and 1 week post-6-OHDA samples, while (b) shows baseline and 12 weeks post-6-OHDA samples. The PCA was performed on all samples using the 50 miRNAs with the largest coefficient of variation based on TMM normalized counts. Each circle represents one sample and each color represents one animal.

Note the data points for Placebo 3 are consistently separated from the other Placebo group points, especially along PC1, which carries over 98% of the variance in both plots.


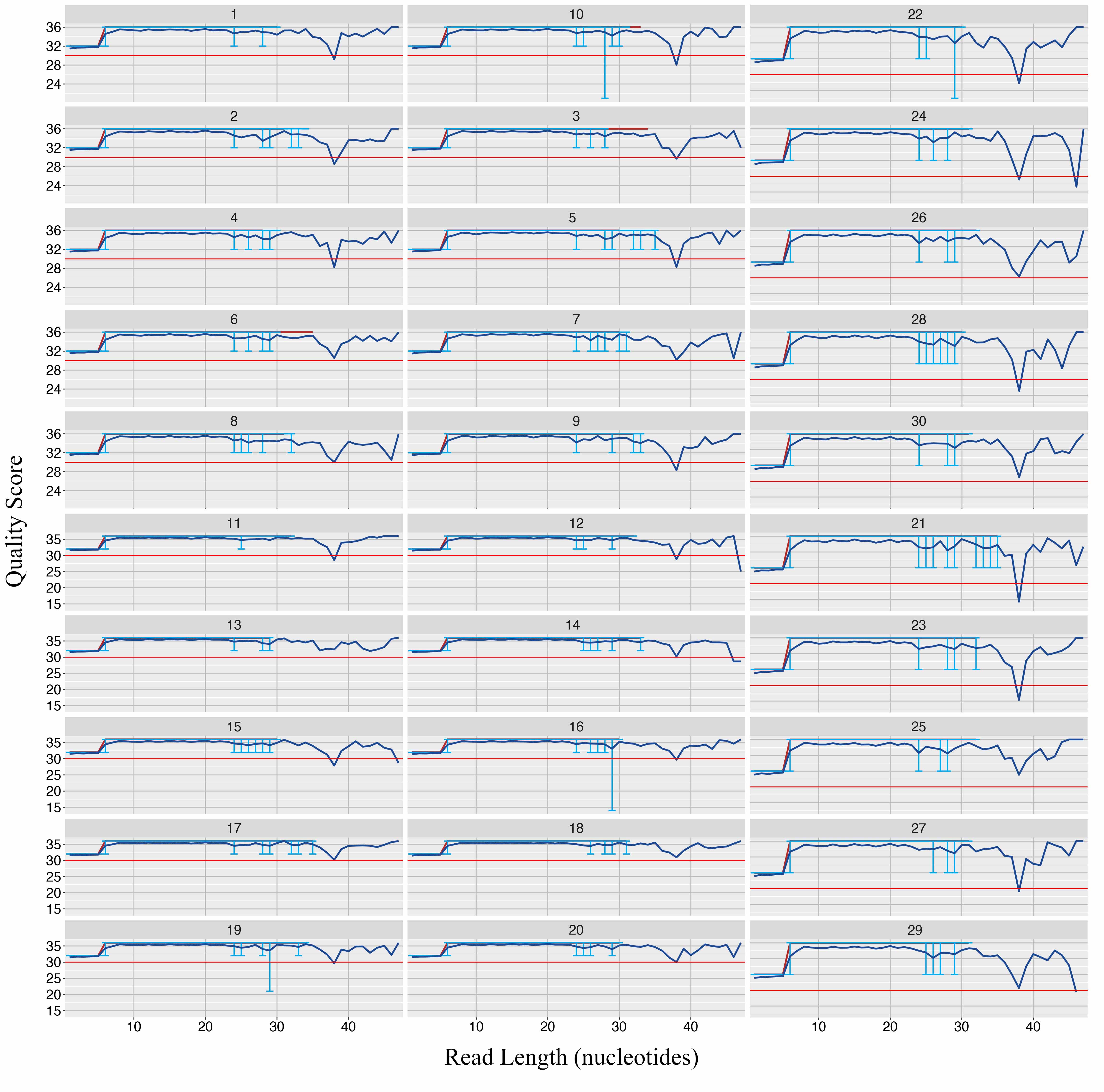


Supplementary Figure 3. Average base quality of the UMI-corrected reads in each of the 30 RNA samples. The position in the read is plotted on the x-axis and the Q-score is plotted on the y-axis. A Q-score of 30 is considered high quality data and represents >99.9% base call accuracy. The dark blue line is the mean value Q-score. The boxplot represents the inter-quartile range, while the whiskers represent the 10% and 90% points.
