# Supplementary material for: Effects of Cardiac Sympathetic Neurodegeneration and PPARγ Activation on Rhesus Macaque Whole Blood miRNA and mRNA Expression Profiles
Source: Biomed Res Int. 2020 May 2;2020:9426204. doi: 10.1155/2020/9426204 (PMC7212295; doi:10.1155/2020/9426204)
Supplement: Supplementary 8 — Supplementary Table 7: summary of sequencing reads and mapping for each sample. [file 9426204.f2.docx]

Supplementary Table 1. Quality and concentration of all 30 extracted total RNA samples. 1wk, 1 week post-6-OHDA; 12wk, 12 weeks post-6-OHDA; RIN, RNA integrity number.

| **RNA Sample** | **Animal** | **Timepoint** | **RIN** | **Concentration (ng/μl)** | **260/280** |
| --- | --- | --- | --- | --- | --- |
| 1 | Pioglitazone 2 | 1wk | 9.1 | 134.4 | 2.08 |
| 2 | Placebo 1 | Baseline | 9.0 | 198.8 | 2.11 |
| 3 | Placebo 1 | 12wk | 9.1 | 119.4 | 2.13 |
| 4 | Pioglitazone 2 | Baseline | 8.7 | 159.2 | 2.11 |
| 5 | Pioglitazone 2 | 12wk | 8.8 | 213.1 | 2.14 |
| 6 | Placebo 1 | 1wk | 8.8 | 170.3 | 2.11 |
| 7 | Pioglitazone 5 | Baseline | 8.9 | 200.9 | 2.10 |
| 8 | Placebo 5 | 1wk | 9.1 | 169.9 | 2.11 |
| 9 | Pioglitazone 5 | 12wk | 8.7 | 221.9 | 2.08 |
| 10 | Placebo 5 | Baseline | 8.7 | 202.9 | 2.08 |
| 11 | Placebo 5 | 12wk | 8.9 | 131.7 | 2.10 |
| 12 | Pioglitazone 5 | 1wk | 8.7 | 338.3 | 2.07 |
| 13 | Pioglitzone 4 | Baseline | 9.2 | 135.4 | 2.06 |
| 14 | Placebo 2 | 1wk | 8.7 | 57.0 | 2.08 |
| 15 | Placebo 2 | 12wk | 8.8 | 75.6 | 2.09 |
| 16 | Pioglitazone 4 | 1wk | 8.9 | 155.2 | 2.11 |
| 17 | Pioglitazone 4 | 12wk | 9.1 | 106.5 | 2.11 |
| 18 | Placebo 2 | Baseline | 8.9 | 52.9 | 2.09 |
| 19 | Pioglitazone 4 | Baseline | 9.1 | 119.5 | 2.10 |
| 20 | Placebo 3 | Baseline | 8.6 | 97.1 | 2.11 |
| 21 | Pioglitazone 4 | 12wk | 9.1 | 258.4 | 2.09 |
| 22 | Placebo 3 | 1wk | 8.6 | 60.5 | 2.08 |
| 23 | Pioglitazone 4 | 1wk | 9.1 | 140.7 | 2.11 |
| 24 | Placebo 3 | 12wk | 9.0 | 94.3 | 2.11 |
| 25 | Placebo 4 | 1wk | 8.8 | 141.3 | 2.09 |
| 26 | Placebo 4 | 12wk | 8.7 | 173.3 | 2.11 |
| 27 | Pioglitzone 1 | Baseline | 9.0 | 200.7 | 2.10 |
| 28 | Pioglitazone 1 | 1wk | 8.8 | 106.1 | 2.09 |
| 29 | Pioglitazone 1 | 12wk | 8.7 | 51.1 | 2.12 |
| 30 | Placebo 4 | Baseline | 8.7 | 157.6 | 2.10 |
