# Supplementary material for: Effects of Cardiac Sympathetic Neurodegeneration and PPARγ Activation on Rhesus Macaque Whole Blood miRNA and mRNA Expression Profiles
Source: Biomed Res Int. 2020 May 2;2020:9426204. doi: 10.1155/2020/9426204 (PMC7212295; doi:10.1155/2020/9426204)
Supplement: Supplementary 9 — Supplementary Table 8: abundance of miRNAs and other small RNAs. [file 9426204.f3.docx]

Supplementary Table 2. List of genes on the RT^2^ Rhesus Inflammatory Cytokine and Receptor PCR Array

| **Position On Array** | **Gene Symbol** | **Position On Array** | **Gene Symbol** |
| --- | --- | --- | --- |
| A01 | AIMP1 | E01 | IL2 |
| A02 | BMP2 | E02 | IL20 |
| A03 | CCL1 | E03 | IL21 |
| A04 | CCL11 | E04 | IL2RA |
| A05 | CCL13 | E05 | IL2RB |
| A06 | CCL17 | E06 | IL2RG |
| A07 | CCL2 | E07 | IL3 |
| A08 | CCL20 | E08 | IL4 |
| A09 | CCL23 | E09 | IL5 |
| A10 | CCL5 | E10 | IL5RA |
| A11 | CCR1 | E11 | IL6 |
| A12 | CCR3 | E12 | IL6R |
| B01 | CCR4 | F01 | IL7 |
| B02 | CCR6 | F02 | CXCL8 |
| B03 | CCR8 | F03 | CXCR2 |
| B04 | CD40LG | F04 | IL9R |
| B05 | CD70 | F05 | IL13RA2 |
| B06 | CSF1 | F06 | LOC710618 |
| B07 | CSF2 | F07 | IL9 |
| B08 | CX3CL1 | F08 | CCL7 |
| B09 | CX3CR1 | F09 | CCL26 |
| B10 | CXCL11 | F10 | LTA |
| B11 | CXCL12 | F11 | MIF |
| B12 | CXCL13 | F12 | NAMPT |
| C01 | CXCL6 | G01 | OSM |
| C02 | CXCL9 | G02 | PF4 |
| C03 | CXCR1 | G03 | SPP1 |
| C04 | CXCR4 | G04 | TNF |
| C05 | FASLG | G05 | TNFRSF11B |
| C06 | IFNA2 | G06 | TNFSF10 |
| C07 | IFNG | G07 | TNFSF11 |
| C08 | IL10RA | G08 | TNFSF13B |
| C09 | IL10RB | G09 | TNFSF14 |
| C10 | IL11RA | G10 | TNFSF4 |
| C11 | IL12B | G11 | VEGFA |
| C12 | IL12RB1 | G12 | XCL1 |
| D01 | IL12RB2 | H01 | ACTB |
| D02 | IL13 | H02 | B2M |
| D03 | IL15 | H03 | GAPDH |
| D04 | IL15RA | H04 | LOC709186 |
| D05 | IL16 | H05 | RPL13A |
| D06 | IL17A | H06 | QGDC |
| D07 | IL17F | H07 | RTC |
| D08 | IL1A | H08 | RTC |
| D09 | IL1B | H09 | RTC |
| D10 | IL1R1 | H10 | PPC |
| D11 | IL1R2 | H11 | PPC |
| D12 | IL1RN | H12 | PPC |

RTC, reverse transcription control; PPC, real-time PCR efficiency control
