# Supplementary material for: Effects of Cardiac Sympathetic Neurodegeneration and PPARγ Activation on Rhesus Macaque Whole Blood miRNA and mRNA Expression Profiles
Source: Biomed Res Int. 2020 May 2;2020:9426204. doi: 10.1155/2020/9426204 (PMC7212295; doi:10.1155/2020/9426204)
Supplement: Supplementary 10 — Supplementary Table 9: mRNA targets of mml-miR-16-2-3p predicted by TargetScan. [file 9426204.f4.docx]

Supplementary Table 3. Sequences of primers used for RT-qPCR

| **Gene Name** | **Protein Name** | **Forward Primer (5' - 3')** | **Reverse Primer (5' - 3')** |
| --- | --- | --- | --- |
| *NFKBIA* | NF-kappa-B inhibitor alpha (IκBα) | GGAGAGTGAGGATGAGGAGAG | TCCAAACACACAGTCGTCATAG |
| *CD36* | Platelet glycoprotein 4 (CD36) | AGTCTCTTTCCTACAGCCCAATG | TGCCACAGCCAGATTGAGAA |
| *STAT1* | Signal transducer and activator of transcription 1 (STAT1) | GAGTTTGATGAGGTGTCTCGGATAG | AACTGTCGCCAGAGAAGATGAA |
| *MAFB* | Transcription factor MafB (MafB) | CTACAAGGTCAAGTGCGAGAAA | GCCACGACTCACAGAAAGAA |
