# Supplementary material for: Effects of Cardiac Sympathetic Neurodegeneration and PPARγ Activation on Rhesus Macaque Whole Blood miRNA and mRNA Expression Profiles
Source: Biomed Res Int. 2020 May 2;2020:9426204. doi: 10.1155/2020/9426204 (PMC7212295; doi:10.1155/2020/9426204)
Supplement: Supplementary 11 — Supplementary Table 10: results of Kyoto Encyclopedia of Genes and Genomes (KEGG) pathway analysis. [file 9426204.f5.docx]

Supplementary Table 4. Differentially expressed small RNAs. Data shown includes results of differential expression analysis including all 10 animals (All animals) and re-analysis without the outlier animal (Placebo 3). TMM, trimmed mean of M values; snoRNA, small nucleolar RNA; miRNA, micro RNA; FDR, false discovery rate.

| **Animals Analyzed** | **Groups compared (Group A v. Group B)** | **Differentially expressed RNA** | **RNA Type** | **Group A TMM** | **Group B TMM** | **Fold Change** | **Uncorrected p value** | **FDR corrected p value** |
| --- | --- | --- | --- | --- | --- | --- | --- | --- |
| **All animals** | 12 Weeks Placebo v. Pioglitazone | *SNORA46* (ENSMMUG00000025257) | snoRNA | 697.62 | 167.57 | -3.89 | 0.0000011 | 0.0006820 |
|  | Placebo Baseline v 12 Weeks | mml-miR-16-2-3p | miRNA | 100.48 | 275.33 | 2.12 | 0.0000010 | 0.0003512 |
|  |  | mml-miR-133d-3p | miRNA | 33.92 | 7.36 | -3.09 | 0.0000023 | 0.0004207 |
|  |  | mml-miR-1262-5p | miRNA | 3.97 | 1.62 | -2.17 | 0.0004667 | 0.0424846 |
|  |  | mml-miR-33b-5p | miRNA | 1.22 | 3.30 | 2.35 | 0.0006497 | 0.0424846 |
|  | Pioglitazone Baseline v 12 Weeks | mml-miR-146b-5p | miRNA | 4.54 | 9.70 | 2.20 | 0.0000418 | 0.0153731 |
|  |  | *SNORD15* (ENSMMUG00000026536) | snoRNA | 1.70 | 5.80 | 2.42 | 0.0000004 | 0.0003415 |
| **All animals except outlier (placebo group animal #3)** | 12 Weeks Placebo v. Pioglitazone | mml-miR-16-2-3p | miRNA | 371.46 | 69.25 | -5.36 | 0.0001555 | 0.0374679 |
|  |  | mml-miR-204-5p | miRNA | 35.53 | 9.53 | -3.72 | 0.0003195 | 0.0384989 |
|  |  | *SNORA46* (ENSMMUG00000025257) | snoRNA | 699.51 | 169.81 | -3.81 | 0.0000211 | 0.0127114 |
|  | Placebo Baseline v 12 Weeks | mml-miR-16-2-3p | miRNA | 114.74 | 326.76 | 2.31 | 0.0000004 | 0.0001542 |
|  |  | mml-miR-133d-3p | miRNA | 40.33 | 8.28 | -3.32 | 0.0000052 | 0.0009051 |
|  |  | mml-miR-1262-5p | miRNA | 4.41 | 1.55 | -2.58 | 0.0000467 | 0.0053706 |
|  | Pioglitazone Baseline v 12 Weeks | mml-miR-146b-5p | miRNA | 4.54 | 9.70 | 2.20 | 0.0000418 | 0.0153731 |
|  |  | *SNORD15* (ENSMMUG00000026536) | snoRNA | 1.70 | 5.80 | 2.42 | 0.0000004 | 0.0003415 |
